# Cognitive Rehabilitation of Acquired Calculation Disturbances

**DOI:** 10.1155/2019/3151092

**Published:** 2019-04-04

**Authors:** Alfredo Ardila, Monica Rosselli

**Affiliations:** ^1^Florida International University, Miami, FL, USA; ^2^Sechenov University, Moscow, Russia; ^3^Florida Atlantic University, Davie, FL, USA

## Abstract

Acalculia is an acquired disorder in calculation abilities, usually associated with left posterior parietal damage. Two types of acalculic disorders are usually distinguished: (1) primary acalculia or anarithmetia, where the patient presents a loss of numerical concepts (difficulties are observed both in oral and written calculations), and (2) secondary acalculia due to a different disturbance in cognition and affecting mathematical abilities. Secondary acalculias are associated with aphasia, alexia, agraphia, executive function disorders, or visuospatial difficulties. This paper is a proposal for clinical intervention to rehabilitation of acquired primary and secondary acalculias.

## 1. Introduction

The disturbance in understanding the numerical system associated with the loss of the ability to perform arithmetical operations is known as *acalculia* (or acquired dyscalculia) [1]. Hence, acalculia is an acquired disturbance, whereas *developmental dyscalculia* or simply *dyscalculia* represents a defect in the ability to normally develop numerical skills [2].

The term “acalculia” was coined by Henschen [3] and defined it as an alteration in mathematical abilities resulting from brain damage. Before Henschen, calculation disturbances were considered to be a component of the acquired language disturbances (aphasias). Later on, Berger [4] proposed a basic distinction between *primary acalculia* and *secondary acalculia*. Ultimately, acalculia can represent a fundamental acquired cognitive disturbance (primary acalculia), but difficulties in performing arithmetical operations can also be found in cases of disturbances in language, attention, reading, writing, and other cognitive impairments (secondary acalculias). Primary acalculia consequently corresponds to a disturbance in understanding numerical concepts and the inability to perform arithmetic operations. Consequently, difficulties are observed both in oral and written calculations. Secondary acalculia is understood as a deficit in numerical abilities due to a different intellectual impairment. Gerstmann [5] suggested that primary acalculia is found associated with agraphia, right-left disorientation, and digital agnosia (inability to recognize the fingers) resulting in a single clinical syndrome that has since become known as “Gerstmann's syndrome” or “angular gyrus syndrome.” Rusconi et al. [6] proposed that Gerstmann's syndrome probably arises from disconnection to separate but colocalized fibre tracts in the subcortical parietal white matter. Contemporary neuroimaging techniques have correlated this syndrome with left posterior parietal lesions [7–9].

McCloskey et al. [10–12] and Caramazza and McCloskey [13] proposed a cognitive model regarding the processing of numbers and the relationship of arithmetical operations. This model includes a distinction between the processing system of numbers (an understanding mechanism and the production of numbers) in addition to the numerical calculation system that includes the necessary processing components to accomplish mathematical operations. In the event of brain injury, these components can be disassociated [14, 15].

Several classifications of acquired calculation disturbances are found in the literature (e.g., [16–19]). Luria [20], for instance, distinguishes three different types of acalculia: optical acalculia, frontal acalculia, and primary acalculia. Hecaen et al. [18] also refer to three variants of acalculia: alexia and agraphia for numbers, spatial acalculia, and anarithmetia or primary acalculia. However, the most frequent distinction found in contemporary literature includes primary acalculia and secondary acalculias.

Ardila and Rosselli [16] proposed a comprehensive classification of acalculias. They distinguished six different types acalculia: (1) primary acalculia or anarithmetia, (2) aphasic acalculia, (3) alexic acalculia, (4) agraphic acalculia, (5) dysexecutive (frontal) acalculia, and finally (6) spatial acalculia. Primary acalculia corresponds to anarithmetia. The remaining types of acalculia are considered as secondary acalculias (Table 1).

A proposal for clinical intervention to rehabilitation of acquired acalculia is presented in this paper. It is important to emphasize that rehabilitation is based in both targeting restitution of the original impaired function and reorganization of functions employing other less or unimpaired cognitive functions [21, 22].

## 2. Cognitive Rehabilitation of Acalculia

Research about acalculia rehabilitation has been limited [23]. Frequently, calculation disturbances are overlooked in cognitive assessments, and numerical ability is sometimes evaluated as a language-dependent ability. However, as in any rehabilitation procedure, the first step is clearly pinpointing the defect characteristics as well as defining the calculation abilities lost or maintained; for instance, sometimes patients with brain pathologies cannot perform arithmetical operations mentally, but they can do in writing; the opposite pattern can also be found. Furthermore, significant variables affecting calculation abilities, such as the education level and the patient's professional activity, must be taken into consideration. The types of errors should be recorded.

In a review of the research devoted to the rehabilitation of acalculia published from 1980 to 2007, only 7 papers were found, all of them single case studies [24]. Regardless of the positive results reported, it is difficult to know which procedure can be considered as the most effective technique in acalculia treatment due to the absence of group studies. For instance, in the Medline review, there were only three papers found reporting the treatment and evolution in acalculia covering the last ten years (2009-2019).

In one of the few studies analyzing the evolution of acalculia, Caporali et al. [25] reported 51 vascular acalculic patients examined at least twice. Results indicated that recovery from acalculia is frequently found during the first months poststroke; furthermore, the initial severity does not significantly influence recovery. Recovery correlates with recovery of auditory comprehension. In a more recent report, Basso et al. [26] examined the relationship between aphasia and acalculia in 98 left brain-damaged patients and the spontaneous recovery from acalculia in 92 acalculia patients. It was found that there is a significant association between aphasia and acalculia; however, 19 patients presented aphasia with no acalculia and six patients presented acalculia with no aphasia. A significant improvement was observed between a first examination carried out between 1 and 5 months postonset and a second examination carried out between 3 and 11 months later.

Few studies have analyzed the brain changes associated with acalculia recovery [27]. Claros-Salinas et al. [28] recorded fMRI in seven patients during calculation and perceptual tasks both before and after acalculia training. Regardless of the heterogeneity of brain lesions associated with acalculia, a common pattern of training-induced changes emerged. Performance improvements were associated with widespread deactivations in the prefrontal cortex. These deactivations were calculation-specific and only observed in patients exhibiting a considerable improvement after training. These findings suggest that the training-induced changes rely on an increase of frontal processing efficiency.

Major strategies used in the rehabilitation of primary and secondary acalculias will be presented. However, frequently cognitive rehabilitation procedures have been implemented in single cases; few studies have included large groups of acalculia patients.

## 3. Anarithmetia Rehabilitation

Primary acalculia is found associated with left posterior parietal lesions [29], usually including the intraparietal sulcus [30]. Tsvetkova [31] suggested that two deficits underlie primary acalculia: (1) an impairment in the spatial perception of numbers and in their mental representation and (2) a deficit in the verbal organization of this spatial perception. These patients present significant difficulties in spatial coordination systems, in other words, in understanding and internally representing spatial language [20, 31]. Several clinical manifestations are observed in cases of primary acalculia, including (1) defects in numerical concepts, (2) difficulties in the comprehension of the position of the numbers within the numerical continuum, for instance, understanding that 12 and 21 are different, (3) impairments in performing arithmetic sequences, for instance, adding or subtracting a certain quantity successively, such as 1, 4, and 7, and (4) defects in arithmetic symbol understanding. Interestingly, these patients also have difficulties with tactile enumeration [32].

It has been observed that anarithmetia usually appears simultaneously with the so-called semantic aphasia [7, 20, 33, 34]; meaning, primary acalculia is associated with disturbances in understanding logical-grammatical relationships. Errors are observed in understanding comparative relations such as “greater than” or “less than,” and therefore, patients cannot determine the magnitude relationship between numbers. For example, they may report that the number 97 is greater than the number 112, because they only consider the numerical value of each digit independently, ignoring the positional value. Regardless that the patients can read digits, their ability to conceptualize numbers is impaired. For example, they cannot recognize the number of dozens or hundreds within a number (e.g., 600) or understand that 30 is composed by 10 + 10 + 10. Simply speaking, anarithmetia is characterized by the loss of understanding of how the numerical system is organized. Departing from this observation, it is easy to conclude that the fundamental aim in primary acalculia rehabilitation is recovering the understanding of numbers and their positional value within the numerical system; that is, numbers represent a quantity, but the quantity that they represent depends on their spatial location.

According to Tsvetkova [31], the initial step consists in organizing sets of elements (for instance, geometric figures) and relating them with a specific numerical value. To reinforce the understanding of quantities, other tasks can be introduced, such as dividing the objects into groups based on a particular feature of the objects, for example, their shape or color and counting the objects in each group while pointing to them. Later on, it is decided how many objects are in the set and writing the number on a paper. Once the acalculia patient has recovered the concept of digits and tens, the rehabilitation will target the concept of numerical composition as well as the relations between numbers; later, the idea of number manipulation through arithmetical operations (adding, subtracting) will be introduced. During this stage, the ordinal position of the numbers and their value depending on their location are explained to the patient and practiced. It is a good idea to initially use concrete elements, for instance, a potato chip, and progressively move to more abstract numerical representations.

Noteworthily, primary acalculia can be observed without language disturbances, but its correlation with aphasia is significant [26]. The rehabilitation of the calculation abilities can be implemented once a level of language understanding and production is acceptable. The recovery of aphasia evidently affects the recovery of calculation abilities. Caporali et al. [25] found that the spontaneous recovery of acalculia in patients with left vascular lesions correlated with the level of recovery in language comprehension.

In conclusion, rehabilitation of the patient with primary acalculia should begin by relearning the basic concepts of number. Later, the patient should move to the acquisition of more sophisticated concepts that imply the relationship between numbers and finally to solving arithmetical operations and numerical problems.

## 4. Secondary Acalculia Rehabilitation

### 4.1. Acalculia Associated with Language Defects

Patients with acalculia due to a language defect (aphasic acalculia) and receiving language therapy usually present a significant improvement in their calculation abilities in a parallel way to the language recovery [35]. In other words, the recovery of calculation disturbances in these patients is parallel to language rehabilitation program. Noteworthily, the calculation deficits are quite different in fluent and nonfluent forms of aphasia, but anyhow, the underlying deficit is the same: a defect in the syntax of the arithmetical operations in Broca's aphasia and a lexical understanding deficit in Wernicke's aphasia [16].

### 4.2. Acalculia Associated with Alexia and Visuoperceptual Defects

Sometimes brain-damaged patients can mentally solve mathematical operations, but they fail in recognizing numbers due to visuoperceptual impairments. The intervention in those cases should be directed to recover the visual perception [36]. Perceptual deficits will impair the performance of numerical tasks that require reading and/or writing. If the ability to write quantities is preserved as it is observed in cases of pure alexia—alexia without agraphia—making the movements as writing numbers in the air will help to its recognition. This same strategy is used to improve the ability to read words and is known as kinetic writing.

The technique known as “number reconstruction” [31] is based on the reconstruction of the number departing from some visual elements, for example, to finish writing numbers whose lines have already been started, such as completing the number 8 starting from number 3. Other strategies can also be used, such as looking for certain elements of a number in another number; for instance, to find the number 1 in the stroke of the number 4. Verbalizing the similarities and differences observed between the visual appearances of different numbers can also be helpful in the reconstruction of the numbers. Spatial orientation tasks, training of right-left dimensions, and analysis and identification of various geometric forms are exercises potentially contributing to the reconstruction of the number representations.

Patients with pure alexia—alexia without agraphia—have difficulties for the visual integration of stimuli (deficit that is known as simultanagnosia) and defects in visual-motor coordination (known as optic ataxia). Consequently, the intervention should include exercises that require the visual exploration of space. Frequently used tasks included in rehabilitation programs suppose a progressive increase in their level of difficulty, beginning with simple movements such as reaching objects, followed by tasks that require more complex movements such as copying figures in two dimensions to conclude with the copy of three-dimensional designs [37]. Training in the reproduction of designs of different shapes, colors, and sizes begin with help from a therapist. For example, a patient is asked to finish copying designs that are partially copied so that they progressively reproduce them completely independently [38].

When there is a visual exploration defect (ocular apraxia) impairing the ability to normally scan quantities, practicing eye-tracking exercises can help to overcome this defect. Rosselli et al. [36] described the rehabilitation of the reading and writing skills of a patient with Balint syndrome, presenting with severe ocular apraxia. Several exercises were used, including visual tracking of objects and working in dozens of trail making tests, specially designed for this patient. In other words, visuokinetic exercises were included in the rehabilitation program designed for this patient. Different letters were presented to him, and he had to reproduce in the air with his hand the movements required to write those letters.

### 4.3. Acalculia Associated with Executive Function Defects

Patients with frontal damage due to diverse conditions, such as head injuries and tumors, usually present executive function disturbances. These patients present particularly significant difficulties in solving mathematical problems. Written calculations are easier than mental calculations. Attentional difficulties significantly contribute to the calculation difficulties. Tsvetkova [31] suggested that providing these patients with attentional control strategies may result in a better attention control and reduction of perseverations. Attentional control strategies include the verbal descriptions of the sequence that is required to successfully solve an arithmetical problem. The patient can be trained to verbalize the operations required and follow them. This is a general procedure that is successfully used with patients with frontal damage and executive function disturbance: to verbalize, as a way to organize and control cognition.

### 4.4. Acalculia Associated with Spatial Defects

Spatial acalculia is usually observed in cases or right hemisphere damage associated with spatial neglect [39]. Spatial neglect refers to the inability to respond to the stimuli presented on the side contralateral to brain pathology (usually the left visual field) and represents a factor impairing the performance of written arithmetical operations. The ability to perform mental operations is much better preserved. Considering that patients with spatial neglect have difficulties to visually explore the surrounding space, several rehabilitation strategies have been proposed, including (1) placing a thick colored mark in the left margin of the sheet of paper and (2) numbering each line to be read. The patient is asked to find the colored mark before beginning to read; using the finger can help to the visual scanning. He has to initially find the numbers located at the beginning of each line. As the treatment advances, the verbal and visual cues are decreased up to the point that the patient is capable to read without the external support. The progressive decrease in neglect is clearly correlated with a decrease in spatial difficulties in reading words and numbers [40].

Rosselli and Ardila [41] described the rehabilitation treatment of a 58-year-old woman who presented alexia, agraphia, and spatial acalculia secondary to a right hemisphere vascular pathology. The rehabilitation program was based on the recovery of spatial neglect and associated spatial difficulties. Initially, the patient could normally perform oral arithmetic operations but was unable to make in written because of the spatial difficulties. In a written mathematical test including addition, subtraction, multiplication, and division, her score was 0/20. The left unilateral spatial neglect, the inadequate mix of procedures, and the impossibility to organize and follow the numbers inside the columns to add them up were very evident. After eight months of therapy, a significant recovery was observed and she was able to perform basic arithmetical operations in writing.

Benavides-Varela et al. [42] reported a large study including 30 right hemisphere-damaged patients and a controlled matched sample. The results showed that patients and controls significantly differ in number comprehension, transcoding, and written operations. Spatial errors were associated with extensive lesions in frontotemporoparietal regions, which frequently lead to neglect. Stepwise regression models consistently revealed that spatial errors were primarily predicted by composite measures of visuospatial attention/neglect and representational abilities. The authors suggest that unilateral right hemisphere lesions can directly affect core numerical/arithmetical processes and that right hemisphere acalculia is not only ascribable to visuospatial deficits as traditionally thought.

## 5. Conclusions

The available information about the cognitive rehabilitation of acquired calculation disturbances is limited. However, some general conclusions can be drawn.

1. In primary acalculia, the fundamental therapeutic goal is to reconstruct the understanding of the numerical system. Patients with primary acalculia seemingly do not understand how the numerical system is organized
2. In aphasic acalculia, numerical impairment and recovery in general parallelize aphasia impairment and recovery
3. When acalculia is due to visuoperceptual disturbances, the fundamental aim of therapy is to recover visual perception
4. In frontal dysexecutive acalculia, providing the patient with external support (e.g., writing) and attentional control strategies (e.g., verbalizing) may result in improving the numerical defect
5. Neglect represents probably not the only but the fundamental defect responsible for the written calculation defects observed in patients with right hemisphere damage. Some simple strategies, such as using clear marks to indicate where to begin to read, can successfully improve the calculation defects

## Figures and Tables

**Table 1:** Classification of acalculias (according to [16]).

Primary acalculia	Anarithmetia

Secondary acalculias	Aphasic acalculia
	*In Broca aphasia*
	*In Wernicke's aphasia*
	*In conduction aphasia*
	Alexic acalculia
	*In central alexia*
	*In pure alexia*
	Agraphic acalculia
	Dysexecutive (frontal) acalculia
	Spatial acalculia

## References

[1] Ardila A. (2018). Acalculia. *Medlink: Neurology*.

[2] Butterworth B., Reed J., Warner-Rogers J. (2008). Developmental dyscalculia. *Child Neuropsychology: Concepts, Theory and Practice. Chichester*.

[3] Henschen S. E. (1925). Clinical and anatomical contributions on brain pathology. *Archives of Neurology and Psychiatry*.

[4] Berger H. (1926). Über rechenstörungen bei herderkrankungen des großhirns. *Archiv für Psychiatrie und Nervenkrankheiten*.

[5] Gerstmann J. (1940). Syndrome of finger agnosia, disorientation for right and left, agraphia and acalculia. *Archives of Neurology & Psychiatry*.

[6] Rusconi E., Pinel P., Dehaene S., Kleinschmidt A. (2010). The enigma of Gerstmann’s syndrome revisited: a telling tale of the vicissitudes of neuropsychology. *Brain*.

[7] Ardila A. (2014). A proposed reinterpretation of Gerstmann’s syndrome. *Archives of Clinical Neuropsychology*.

[8] Ardila A., Concha M., Rosselli M. (2000). Angular gyrus syndrome revisited: acalculia, finger agnosia, right-left disorientation and semantic aphasia. *Aphasiology*.

[9] Ardila A., Rosselli M. (1990). Acalculias. *Behavioural Neurology*.

[10] McCloskey M., Caramazza A., Basili A. (1985). Cognitive mechanisms in number processing and calculation: evidence from dyscalculia. *Brain and Cognition*.

[11] McCloskey M., Sokol S. M., Goodman R. A. (1986). Cognitive processes in verbal number processing: inference from the performance of brain-damaged subjects. *Journal of Experimental Psychology: General*.

[12] McCloskey M., Aliminosa D., Macaruso P. (1991). Theory based assessment of acquired dyscalculia. *Brain and Cognition*.

[13] Caramazza A., McCloskey M., Deloche G., Seron X. (1987). Dissociation in calculation processes. *Mathematic Disabilities: A Cognitive Neuropsychological Perspective*.

[14] Dagenbach D., McCloskey M. (1992). The organization of arithmetic facts in memory: evidence from a brain-damaged patient. *Brain and Cognition*.

[15] Pesenti M., Seron X., Van Der Lider M. (1994). Selective impairment as evidence for mental organisation of arithmetical facts: BB, a case of preserved subtraction?. *Cortex*.

[16] Ardila A., Rosselli M. (2002). Acalculia and dyscalculia. *Neuropsychology Review*.

[17] Grafman J., Boller F., Grafman J., Rizzolatti G., Goodglas H. (1988). Acalculia. *Handbook of Neuropsychology (Vol. 1)*.

[18] Hecaen H., A ngelergues T., Houiller S. (1961). Les varietes cliniquesdes acalculies au cours des lesions retrorolandiques. *Revue de Neurologie*.

[19] Levin H., Goldstein F. C., Spiers P. A., Heilman K. M., Valenstein E. (1993). Acalculia. *Clinical Neuropsychology*.

[20] Luria A. R. (1977). *Las Funciones Corticales Superiores en el Hombre*.

[21] Willmes K., Goldenberg G., Miller B. L. (2008). Acalculia. *Handbook of Clinical Neurology, Vol 88 (3rd Series) Neuropsychology and Behavioral Neurology*.

[22] Zaunmüller L., Domahs F., Dressel K. (2009). Rehabilitation of arithmetic fact retrieval via extensive practice: a combined fMRI and behavioural case-study. *Neuropsychological Rehabilitation*.

[23] Cappelletti M., Cipolotti L. (2010). *The Neuropsychological Assessment and Treatment of Calculation Disorders*.

[24] Basso A., Cattaneo S., Girelli L. (2011). Treatment efficacy of language and calculation disorders and speech apraxia: a review of the literature. *European Journal of Physical and Rehabilitation Medicine*.

[25] Caporali A., Burgio F., Basso A. (2000). The natural course of acalculia in left-brain-damaged patients. *Neurological Sciences*.

[26] Basso A., Caporali A., Faglioni P. (2005). Spontaneous recovery from acalculia. *Journal of the International Neuropsychological Society*.

[27] Bernal B., Ardila A., Altman N. R. (2003). Acalculia: an fMRI study with implications with respect to brain plasticity. *International Journal of Neuroscience*.

[28] Claros-Salinas D., Greitemann G., Hassa T. (2014). Neural correlates of training-induced improvements of calculation skills in patients with brain lesions. *Restorative Neurology and Neuroscience*.

[29] Grimaldi M., Jeanmonod R. (2018). Acute stroke presenting with isolated acalculia. *The American Journal of Emergency Medicine*.

[30] Dehaene S., Molko N., Cohen L., Wilson A. J. (2004). Arithmetic and the brain. *Current Opinion in Neurobiology*.

[31] Tsvetkova L. S., Ostrosky F., Ardila A., Dochy R. (1996). Acalculia: aproximación neuropsicológica al análisis de la alteración y la rehabilitación del cálculo. *Rehabilitación neuropsicológica*.

[32] Cohen Z. Z., Arend I., Yuen K. (2018). Tactile enumeration: a case study of acalculia. *Brain and Cognition*.

[33] Ardila A. (1993). On the origins of calculation abilities. *Behavioural Neurology*.

[34] Ardila A., López M. V., Solano E., Ardila A., Ostrosky-Solis F. (1989). Semantic aphasia reconsidered. *Brain Organization of Language and Cognitive Processes*.

[35] Basso A. (2003). *Aphasia and Its Therapy*.

[36] Rosselli M., Ardila A., Beltran C. (2001). Rehabilitation of Balint’s syndrome: a single case report. *Applied Neuropsychology*.

[37] Sohlberg M. M., Mateer C. A. (2017). *Cognitive Rehabilitation: An Integrative Neuropsychological Approach*.

[38] Ben-Yishay Y. (1983). *Working Approaches to the Remediation of Cognitive Deficits in Brain Damaged Persons. Rehabilitation Monographs*.

[39] Ardila A., Rosselli M. (1994). Spatial acalculia. *International Journal of Neuroscience*.

[40] Ardila A., Rosselli M. (2007). *Neuropsicologia Clinica*.

[41] Rosselli M., Ardila A., Ostrosky F., Ardila A., Dochy R. (1996). Rehabilitación de la alexia y la agrafía. *Rehabilitación neuropsicológica*.

[42] Benavides-Varela S., Piva D., Burgio F. (2017). Re-assessing acalculia: distinguishing spatial and purely arithmetical deficits in right-hemisphere damaged patients. *Cortex*.

